# Cancer therapy: know your enemy?

**DOI:** 10.1186/s40348-014-0010-2

**Published:** 2014-10-31

**Authors:** Mike-Andrew Westhoff, Oliver Brühl, Klaus-Michael Debatin

**Affiliations:** Department of Pediatrics and Adolescent Medicine, University Medical Center Ulm, Eythstrasse 24, 89075 Ulm, Germany; Laboratorio Analisi Sicilia Catania, 96016 Lentini (SR), Italy

**Keywords:** Tumor microenvironment, Adhesion-mediated apoptosis resistance, Tumor-associated macrophages

## Abstract

**Background:**

Most cancer therapies are devised for adult or even elder patients. However, when dealing with pediatric cancers, additional considerations are needed.

**Conclusions:**

This review discusses non-classic components of tumors and highlights possible treatment approaches which might be of particular benefit for children and adolescents.

## Introduction

Cancer is still foremost to be considered a disease of old age, generally occurring after a lifetime of accumulated insults to our DNA's integrity which finally overcome the body's repair and control mechanisms; indeed, over a third of all cancer cases occur in members of the population who are 75 years of age or older, while less than 1% can be found in children and adolescents [1]. This, in turn, means that most therapies have been developed and optimized towards adult and elderly patients in particular and that most data on efficacy and long-term effects are also derived from these populations.

A distinct set of problems and deliberations has to be considered when designing novel treatment strategies in pediatric oncology. Foremost among those are the long-term adverse effects of treatment, which might only occur decades after the initial disease has been successfully treated, these include among others peripheral neurotoxicity [2], cardiovascular disease, and second malignant neoplasms [3]. For example, already 1 year after radiotherapy, the risk of acute leukemia and non-Hodgkin lymphoma is increased in patients with solid tumors [4]. So far, comprehensive studies addressing these issues are sadly lacking. In addition, there are rather acute effects of treatment that can severely affect a child's quality of life, and among these can be treatment-induced loss of hearing, fatigue and weakness, increased susceptibility to infection, and growth retardation. Current treatment options often appear harsh and even morbid to young patients and their parents. There are several recent cases where the patients and/or their guardian have opted out of a successful treatment plan and sought ‘alternative’ options, often with unfortunate consequences^a^. Finally, while an unsurpassed success over the last 30 years has been achieved in the therapy for childhood leukemias, pediatric cancer is the most common cause of death in children (aged 1 to 14 years) and among the four most common forms of deaths in teenagers and young adults (aged 14 to 25) [1]. Therefore, novel therapeutic approaches, taking into account the specific difficulties regarding the treatment of children and young adolescents, are clearly needed.

While we argue elsewhere that chronification of the malignancy can be a valid approach in older or elderly cancer patients [5], especially since the long-term effects of treatment, which might only manifest several decades later, do not need to be taken into account, we do not have this luxury when dealing with children or adolescents. Here, both the long-term impact on the patient's health from excessive treatment toxicity, as well as the totality of life lost if a too weak chemo- or radiotherapy is selected must be taken into account. Therefore, increased treatment specificity for the whole of the cancerous cell population, while concurrently minimizing the collateral damage to healthy tissue, is the paramount challenge when devising novel treatment options for young patients.

The first question for this strategy to succeed that needs to be answered seems almost trivial, however, the last decade or so has demonstrated time and time again that our textbook knowledge is far from complete. This question is: What is the sum total of the tumor?

## Cancer as a genetic disease

The modern view of what constitutes cancer evolved over the last 60-odd years, beginning in 1953 with the multiple-hit hypothesis, which led to the identification of oncogenes and proto-oncogenes in the 1970s and the subsequent development of the cancer progression, or multistep carcinogenesis, model in 1990 [6]: an individual cell, or a small population of cells, accumulates an increasing amount of mutations over time, which leads to uncontrolled growth, an ever increasing independence from the surroundings and finally, the ability to avoid death signals and to metastasize - the so-called hallmarks of cancer [7]. This model suggests that the phenotype of a cancer is due to a combination of essential driver mutations, those which make tumor progression possible, and random passenger mutations. If a mutation occurs, either due to the inherent genetic instability of cancer cells or provoked by treatment, that gives a cancer cell a growth advantage for the present fitness landscape that leads to a clonal expansion of this subpopulation [5]. The cancer stem cell (CSC) model, which, although proposed in 1937, only gained traction in the 1990s [6], adds another layer of complexity. These mechanisms are still valid for the cancer cell of origin, which can - but need not be - identical to the CSC. The CSC hypothesis postulates the existence of a stem cell niche within the tumor where a cell population resides that has acquired properties associated with normal stem cells, such as self-renewal and differentiation into multiple cell types found within the tumor. CSC make up only a fraction of a percent of total tumor bulk, and due to the high plasticity of their phenotype and their ability to remain quiescent for probably many decades, they are held responsible for relapse and therapy failure [6,7].

In essence, the prevalent, but incomplete, view is of cancer as a genetic disease caused by the accumulation of mutations. Therefore, using tools and models provided by the fields of (population) genetics and ecology, the failure to treat a cancer successfully over a prolonged period of time can best be understood as our failure successfully to compete in the Red Queen arms race^b^: because of the genetic instability inherent in cancer cells, we are targeting a rapidly changing collective of populations that quickly adapt to changes in the fitness landscape with a mechanistically very limited arsenal of tools. In essence, both radio- and the majority of chemotherapy function by either directly or indirectly inducing DNA damage and, therefore, to a mutational change within the cancer cell that allows propagation despite compromised DNA integrity. This could cause, for example, the deactivation of cell cycle checkpoints or enhanced exclusion of therapeutic reagents or increased expression of multidrug-resistance proteins, leading, as the name of the latter example already suggests, to resistance not only against already used therapeutic avenues but also against possible future approaches. So far, frequently, the only available response to emerging resistance has been the increase in ‘firepower’, i.e., enhanced doses of the therapeutics. Taking our cues from the field of population genetics, two possible approaches can be identified that might help to treat the patients more successfully, i.e., keep up in the arms race. One, increase the arsenal at our disposal to include treatment options which kill the cancer cells not by direct DNA damage, but alternative routes, for example, triggering of death receptors via synthetic ligands or antibodies [8]. Two, change the target to something that, taking up the Red Queen metaphor, runs more slowly, i.e., target a genetically more stable component of the tumor, for example, the tumor microenvironment (TME) or the interface between mutated cancer cell and TME. Here, we will focus on this latter aspect.

## The tumor microenvironment

In 1889, Paget suggested in what has become known as soil-and-seed hypothesis that a cancer cell thrives wherever it encounters a permissive environment, thus explaining the preferred sites of metastasis [6]. While the ensuing research has focused mainly on the seed, i.e., the mutated cancer cell, emerging evidence suggests that the soil, i.e., the microenvironment, far from being a randomly encountered niche during metastasis, is, already at the site of the primary tumor, a specialized tissue that should be viewed as an integral part of the tumor [7]. While the general focus when dealing with the TME lies on the tumor-associated stroma cells, it is composed of not only cells, such as fibroblasts, epithelial and endothelial cells, macrophages, and leukocytes, but also components of the extracellular matrix, such as collagen and fibronectin [9]. Mutated cells communicate with these components via heterotypic cell-cell interaction, in the case of other cells, or via cell-substrate interaction, when dealing with components of the extracellular matrix [5]. These interactions are not only essential for the three-dimensional organization of the tumor, including but by no means limited to vascularization, they also mediate a phenomenon called AMAR, or adhesion-mediated apoptosis resistance [5]: cancer cells which are in contact with their microenvironment show increased resistance towards both radio- and chemotherapy, which induce apoptosis, a form of cell suicide. This is mediated by enhanced survival signaling or a reduction of the death signaling and, importantly, specifically blocking these interactions can in turn sensitize the mutated cells for apoptosis, even if the cell and its TME are not physically removed from each other [5]. Therefore, inhibiting the interaction between mutated cell and TME, in essence, blinding the cancer cell to its surroundings, leads to a reduction in therapeutic intervention needed, thus lowering the risks of long-term side effects. Furthermore, increased isolation from the cancer niche might even lead to anoikic cell death, the cancer cells dying without the need of additional therapeutic intervention [5]. It has even been suggested that the development of the TME occurs concurrently and is essential for the multistep carcinogenesis [10] and there is compelling evidence for the formation of a premetastatic niche prior to the establishment of a colony of mutated cancer cells, i.e., the soil is fertilized prior to the seed growth [11]. Therapeutically, reestablishing the ‘normal’ unaltered microenvironment can be sufficient to block tumor progression and tumor formation [12,13]. In addition, far from being a hindrance during local invasion and metastasis, which needs to be broken down in order for the cancer cell to progress to distant sites, several components of the TME actively cooperate with tumor cells in these processes [9]. Our own recent work suggests that by inhibiting the interaction between cancer cell and TME - in particular, Fibronectin, which was predominately produced by those cells at the leading invasive edge of the tumor, inhibits their migratory capacity - metastasis can be blocked (summarized in [5]). However, depending on the target, the opposite can occur, blocking desmosomes, tethering point for intermediate filaments, or tight junctions, at the boundary between apical and basal domains of the plasma membrane, within the tumor can actually increase invasion [5].

The complexity of the TME's role has been further highlighted in recent research into the role of macrophages within the tumor. These cells, which are phagocytes involved in both innate and adaptive immunity, make up a rather mobile component of the TME. They are attracted to hypoxic and necrotic areas of the tumor and are believed to mediate chronic inflammation, apoptosis resistance, and neovascularization, i.e., to ensure tumor survival and propagation. Tumor-associated macrophages (TAMs) can often not penetrate the tumor growth due to the altered metabolism of the cancer cell, which, in essence, creates a too toxic environment for TAMs to thrive [14]. Basically, the same signals which attract TAMs to the tumor prevent them from reaching the majority of cancer cells. This seems rather counter-intuitive if TAMs are recruited to the tumor to facilitate oxygen supply and, thus, metabolic alterations and survival of the tumor. However, if one considers the role of macrophages in the body's immune response, a rather intriguing possibility emerges: while the TAMs can be co-opted by the cancer cells at the tumor periphery to mediate invasion, they need to be kept out of the actual growth, as they might exert an anti-tumorigenic effect there. Several new studies seem to confirm that, both a healthy bacterial flora to activate the immune system and macro-phages to execute the immune response are needed for chemotherapeutic intervention to be fully successful [15-17]. Interestingly, therapeutically altering the TME to enhance the macrophage response also seems practical in non-solid cancers, such as leukemia [15].

A frequently voiced objection to targeting the TME is that it provides a far less comfortable therapeutic window, i.e., the view that non-mutated cells within a cancer are patient cells and therefore targeting those cells will lead to increased damage of healthy tissue. While correct on a strictly genetic level, this *caveat* does not take into account the dynamic reciprocity that exists between the different components of the tumor. In essence, not only does the TME provide the mutated cell with a niche in which to survive, but the cancer cell also alters the TME, giving it a distinct phenotype and individual epigenetic signature [9,18]. Importantly, unlike mutated cancer cells, cells from TME readily revert to their non-disease associated phenotype, while providing a slower proliferating, genetically more stable target for therapeutic interventions compared to cancer cells [18]. Therefore, blocking the influence of the cancer cells on the TME and of the TME on the cancer cells, in essence, either targeting the soil or the seed's ability to interact with it, should lead to a more localized disease and can be sufficient to kill cancer cells, either by needing a much reduced amount of additional therapy or without the need of any further radio- or chemotherapy.

## Conclusions

While pediatric cancers are a relatively rare form of this malignancy, they are among the leading causes of death in children and adolescents. When considering quantity of life lost in years, it is difficult to imagine a more deadly disease. However, most therapeutic approaches are devised focusing adult or even elderly patients, thus often not considering the long-term burden on the patients' health. Most therapies are based on the maximum dose density of the Norton-Simon model, which postulates maximal killing of tumor cells by using therapeutic doses close to, or in extreme cases exceeding the limit of tolerable [19]. This approach, if not instantaneously successful, leads to an arms race that frequently ends with the emergence of a therapy-resistant tumor. On the other end of the spectrum is the adaptive therapy approach, which postulates a chronification of the disease [19], while we favor this approach in general [5], it is not feasible when treating pediatric cancers. A third option, metronomic therapy which postulates continuous treatment with lower therapeutic doses, might have less severe long-term side effects, but is not designed to prevent emergence of resistance [19].

**Figure 1:**
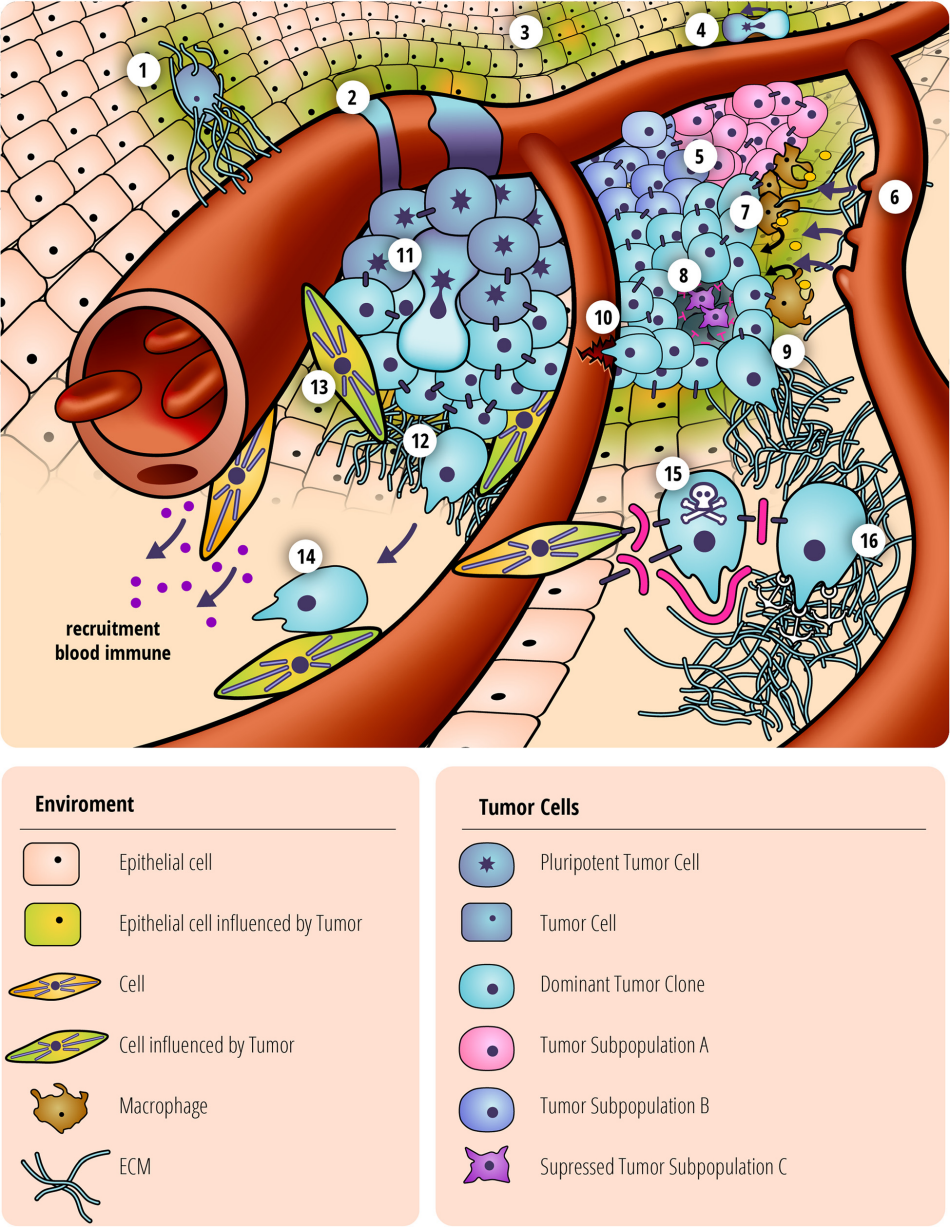
**Tumor composition.** Generalized schematic representation of a tumor, showing both components that make up the malignancy: Mutated tumor cells, both putative tumor stem cells in their niche (11) and various differentiated subclonal populations (5), which can be in direct competition (8). The microenvironment, composed of newly formed (2) and forming (6) blood vessels, components of the extracellular matrix, such as fibronectin (9), normal surrounding tissue, and cells altered by their proximity to the mutated tumor cells, including fibroblasts (13). Of note, the highly motile TAMs (7), which do not penetrate the malignancy deeply, but at the tumor periphery can either facilitate tumor growth and invasion or enhance the chemotoxic effects of therapy. Several forms of interaction between mutated tumor cell and microenvironment are also shown, such as the destruction of the microenvironment during invasion (12), the co-opting of blood vessels to increase dissemination (10), and the creation of a novel microenvironment during invasion (1), establishing a premetastatic niche prior to its colonization by cancer cells (3) and the possible formation of distant metastasis by de-differentiation (4). This view of cancer leads to additional potential therapeutic avenues to pursue [5], such as the possibility of blocking the tethering points between cancer cell and matrix and the communication between mutated cancer cells and tumor-associated cells, which can lead to reversion of the tumor microenvironment to ‘normal’ tissue that does not necessarily support tumor growth and either death of the tumor cell by a specialized form of cell death (anoikis) (14) or at least its sensitization towards conventional therapy, i.e., the inhibition of AMAR. Importantly, therapeutic targets must be carefully chosen, as not to enhance cell motility and thereby increase potential invasion/metastasis.

To devise new, successful treatment options for pediatric cancer, we postulate going back to the stratagem first suggested by the Chinese strategist Sun-Tzu more than 2,000 years ago: The success in a campaign can be greatly enhanced, if one knows not only oneself but also one's opponent. In the case of cancer, we have just begun to address the latter aspect. By understanding cancer not just as a population of mutated cells but as a complex ecosystem consisting of mutated cells, non-mutated but epigenetically altered cells, unaltered cells and extracellular components, new modes of intervention can become apparent (Figure 1). For example, isolating the mutated cancer cell from it's surrounding, either by targeting the TME or the cells' interaction with the TME leads to three distinct therapeutic benefits.

1. AMAR is reduced, i.e., lower doses of therapeutic reagents are needed to kill the same amount of cancer cells; therefore, side effects and risks of secondary cancers are reduced. Ideally, this reduction leads to…
2. Total isolation of cancer cells from their TME and each other (sometimes rather whimsically referred to as Alcatraz approach), which can lead to anoikis, i.e., the death of tumor cells without additional therapeutic intervention. Even if these points are not fully achieved, they can still contribute significantly to…
3. Reduced invasion and metastasis. The interaction between tumor cell and TME is essential for the spread of cancer. Even if mutated cancer cells do not die via this intervention, cancer still potentially becomes a more localized disease. This in turn opens additional therapeutic options to be pursued.

The role of the TME is far from being fully understood, and further research is clearly needed, as seen for example, when discussing the TAMs. Here, the complex interaction between bacteria, immune system, and therapy clearly indicates that individual components of the TME can have diverse, apparently contradictory functions. Importantly, understanding these interactions has direct implications for patient care. In this particular case, the routine administration of antibiotics in immunocompromised cancer patients, while avoiding potential infections, reduces the efficacy of chemotherapy.

## Footnotes

^a^Examples, such as the cases of Daniel Hauser or Sarah Hershberger, are discussed by surgical oncologist David H. Gorski at www.sciencebasedmedicine.org.

^b^The Red Queen hypothesis postulates antagonistic coevolution between competing populations (either of the same species or of distinct species, e.g., predator/prey or host/parasite). For the relationship between those populations to remain in equilibrium, both have to constantly adapt and evolve in an unstable environment.
